# Needleless Ablation of Osteoid Osteoma and Osteoblastoma: The Emergent Role of MRgFUS

**DOI:** 10.3390/jcm11010128

**Published:** 2021-12-27

**Authors:** Flavia Cobianchi Bellisari, Pierpaolo Palumbo, Carlo Masciocchi, Carmine Zoccali, Antonio Barile, Francesco Arrigoni

**Affiliations:** 1 Department of Biotechnological and Applied Clinical Sciences, University of L’Aquila, 67100 L’Aquila, Italy; carlo.masciocchi@univaq.it (C.M.); antonio.barile@univaq.it (A.B.); arrigoni.francesco@gmail.com (F.A.); 2 Abruzzo Health Unit 1, Department of Diagnostic Imaging, Area of Cardiovascular and Interventional Imaging, 67100 L’Aquila, Italy; palumbopierpaolo89@gmail.com; 3 Department of Oncological Orthopaedics, IFO-Regina Elena National Cancer Institute, 00144 Rome, Italy; carminezoccali@libero.it; 4 Department of Anatomical, Histological, Forensic Medicine and Orthopaedic Science, University of Rome, 00185 Rome, Italy

**Keywords:** osteoid osteoma, osteoblastoma, MRgFUS, interventional radiology, benign bone tumors

## Abstract

Osteoblastoma (OB) and osteoid osteoma (OO) are benign bone-forming tumors, with nearly identical basic microscopic features. The main difference is dimension (OO has usually a nidus measuring <2 cm in diameter). In addition, OB is biologically more active than OO, with a tendency to grow in size. Historically, treatments have included surgical resection and analgesics, although invasiveness and poor tolerance have led to the current standard of care moving toward interventional radiology, where radiofrequency ablation (RFA) represents the most diffuse technique. Magnetic resonance-guided focused ultrasound surgery (MRgFUS) has recently emerged as another innovative alternative treatment, providing tumor ablation through a needleless and ionizing radiation-free modality. In addition, this technique has the ability to guarantee a very precise and controlled increase in temperature, delivering small amounts of energy that can accurately destroy only the lesion, avoiding healthy surrounding tissues. The present review focuses on MRgFUS as the less invasive, safe, effective, and durable treatment option for the management of osteoid osteoma and osteoblastoma, including a description of technical details, indications and outcomes.

## 1. Introduction

### 1.1. Tumors and Non-High Intensity Focused Ultrasound (HIFU) Technique

Osteoid osteoma (OO) and osteoblastoma (OB) are rare, benign, bone-forming tumors that account for about 12% and 3%, respectively, of all benign bone tumors [1]. Although these tumors share histological features [2,3], they are two distinct entities differing in dimensions, topographic distribution, clinical and imaging presentation and potential for progression [4].

OO usually affects patients in the first two decades of life (male to female ratio 3:1). Common sites are long tubular bones, typically the diaphysis of femur and tibia [5]. The most used classification divides them into cortical, medullary (or endosteal) and subperiosteal: cortical is the most common, followed by medullary, while subperiosteal is the least common type. Usually intra-articular lesions, while quite rare, may be difficult to detect [6]. The main symptom is pain that flares up nocturnally. When intraarticular, a functional limitation is frequent [5].

Computed tomography (CT) is the imaging modality of choice in the demonstration of the radiolucent tumor nidus. It is possible to also see a variable amount of central nidus mineralization and surrounding reactive sclerosis. CT is also fundamental in detecting small lesions, which could be obscured by overlying sclerosis on radiographs [7].

Magnetic resonance (MR) is helpful in showing the reactive bone marrow edema around the nidus, demonstrating an increased T2-weighted signal intensity. This is of paramount importance when looking for the nidus that can be extremely small and difficult to find even on the CT [6].

Bone scintigraphy may be helpful in case of mild symptoms, illustrating an overall marked increased activity. A characteristic “double density” sign is often described due to a higher concentration of radionuclide in the nidus than in the surrounding reactive sclerosis and cortical thickening [8].

Usually, OB has a nidus of diameter >2 cm with less pronounced surrounding osseous reaction and is biologically more active than OO [9]. OB commonly arises in the posterior aspects of the spine, and in long bones of the lower extremities [10].

Clinically, the main symptom is pain, usually continuous and generally without nocturnal flares; moreover, there is a lower response to salicylates than in OO. Spinal or costovertebral lesions may be associated with scoliosis and may occasionally present with neurologic symptoms due to epidural or paraspinal extension.

Upon imaging, OBs present various characteristics including focal osteolysis, osteosclerosis or mixed pattern. Though OB may resemble OO, the nidus is usually larger (>2 cm in diameter) and the surrounding osseous reactions and scleroses are less pronounced [11].

The natural course of these tumors is still unknown, and the goal of the treatment is to remove the nidus to alleviate pain. A natural course with spontaneous resolution of OO has been described, but long-term medication therapy is not acceptable, especially in young people [12]. Non-operative treatment has the potential side effects of protracted nonsteroidal anti-inflammatory medications (NSAIDs); furthermore, the possible progression to OB with prolonged NSAIDs [13] has been described.

Traditional management of these tumors is surgical excision, which is associated with a significant morbidity and difficulty in performing a complete resection, mostly on highly vascularized tumors. Moreover, the reduced dimension of OO makes its localization difficult with traditional surgery and increases the risks for relapse due to incomplete excision [14].

To avoid drawbacks related to surgery, in recent years, minimally invasive techniques emerged and CT-guided radiofrequency ablation (RFA), since it was first reported to be successful by Rosenthal et al. is now accepted as the gold standard [15].

RFA is performed with a multi-detector CT and multiplanar reconstructions are used to depict the optimal path to cover the whole nidus. This technique gives a great precision in delivering energy for thermal ablation, protecting tissues surrounding the target area; it has been reported to be a safe technique with a superior long-term efficacy and an important reduction in hospitalization cost and duration when compared to other surgical techniques [16].

Recent literature reported a success rate of this procedure close to 100% and a recurrence of about 5% with a very low rate in post-procedural complications [17,18].

Another technique used for the ablation, especially of OOs, is laser. Its effectiveness has been largely described in literature. Gangi et al. found a 98% rate of primary success 2 weeks after treatment and only six cases of recurrence with a successful re-treatment. One of its main drawbacks, however, is that it is possible to induce necrosis in quite small areas, representing an important limit to this technique [19].

Microwave ablation (MWA) is another alternative in the field of interventional radiology. In this procedure, energy is delivered in the body through a needle and coagulation necrosis is obtained thanks to heat produced by the agitation of molecules. This type of ablation propagates energy through all types of tissues, producing a higher direct heating than other techniques, resulting in more powerful high vascularized structures or vascular bundles close to the target. However, there has been a drop in using this modality of treatment, as one of the main drawbacks is the ellipsoid shape of the ablated area, not optimal in the treatment of this type of lesions. The costs are usually higher compared with RFA [20].

### 1.2. Beyond Standard Techniques

Percutaneous ablation techniques, especially CT-RFA, are nowadays the first-line option for these benign bone lesions [21].

Although this technique is minimally invasive, there are some potential complications that may occur during needle passage (e.g., broken drill, infection, cellulitis, sympathetic dystrophy). Other complications include thermal damage to the vascular, nervous, and articular structures (cartilage, capsule, ligaments, tendons) and soft-tissue burns, mostly skin burns [22].

Needle positioning during CT-RFA may be challenging and in case of larger lesions (>10 mm) multiple treatments or consecutive ablations during the same session may be required [23].

In addition, even at the price of a higher radiation exposure for the patient and the operator, it is possible to perform a real-time guidance due to the intrinsic limit of CT, it is not possible to have a real-time control of the temperature in the region of treatment [24].

In this scenario, a novel radiation-free technique of thermal ablation is represented by magnetic resonance-guided focused ultrasound surgery (MRgFUS) that is reported to have similarly high rates of success and nearly no complications [6,25,26]. Furthermore, MRgFUS allows for avoidance of partial treatments encountered with CT-RFA due to inaccurate needle positioning [12].

### 1.3. MRgFUS Technique

MRgFUS is a novel non-invasive procedure, which allows for ablating bone lesions without even touching the skin of the patient, and without antibiotic prophylaxis or radiation exposure. This revolutionary system uses the thermal ablation power of ultrasound (US) combined with the radiological guidance of MR [25].

The US beam is focused on the lesion and generates sufficient heat (57 °C for at least one second) to induce coagulative necrosis and cell death with minimal heat generation around the target. Treatment consists in a series of “sonications”, where this term indicates the act of delivering an amount of high-intensity sound waves to agitate matter and it is used to describe each energy deposition within a focal point into the region of interest. The procedure involves some preliminary low-energy sonications that are used as a preliminary test to verify the correct position of the target area and the correct focalization of the US beam within the target area before starting with the full-energy ablations. Each sonication usually lasts for a few seconds, ablating few cubic millimeters of lesion [27].

The procedure is performed under MRI guidance through which the operator can accurately evaluate the anatomy of the region. This is particularly helpful, allowing a meticulous evaluation of both target lesion and surrounding structures throughout the treatment. In addition, the system allows a real-time control of the temperature thanks to a thermometry using specific sequences, proton resonance frequency (PRF), and by detecting all temperature-dependent MR phase variables, showing areas where the temperature is high enough to generate coagulation necrosis. The temperature increase is measured on the adjacent peri-skeletal tissue. In fact, due to the absence of moving protons in the cortical zone, PRF sequence is not able to detect temperature on bone surface, whereas it reveals a temperature increase which propagates by conductive processes from the bone to the adjacent tissues. Prior to treatment, the patients were submitted to MR imaging to determine their eligibility and plan the treatment. In fact, it is fundamental to evaluate accessibility to the lesion [28].

Acoustic attenuation in bone is 30–60 times higher than in soft tissue; therefore, the lesions that benefit from MRgFUS are the most superficial ones [26]. Lesions deep in the bone more than few millimeters are not suitable to be treated directly [29]. However, due to the high attenuation of the bone surface, it is possible to perform a pain palliation through the ablation of the periosteal surface. At the same time, other bone segments, metal devices or scars that are between the skin and the lesion can interfere with the correct focalization of the US beam [30].

Another important factor is the distance of the lesion from the skin surface, because due to the interposition of soft tissues, it is possible that the US beam loses its effectiveness. If the distance between the skin surface and the target lesion exceeds 10 cm, the ablation can be compromised [26].

OOs are mostly located superficially (at cortical or subendosteal level) and in the appendicular skeleton (50% at femoral and tibial level); this is an important advantage as it can be easily reached and is far from a sensitive structure [30].

**Figure 1:**
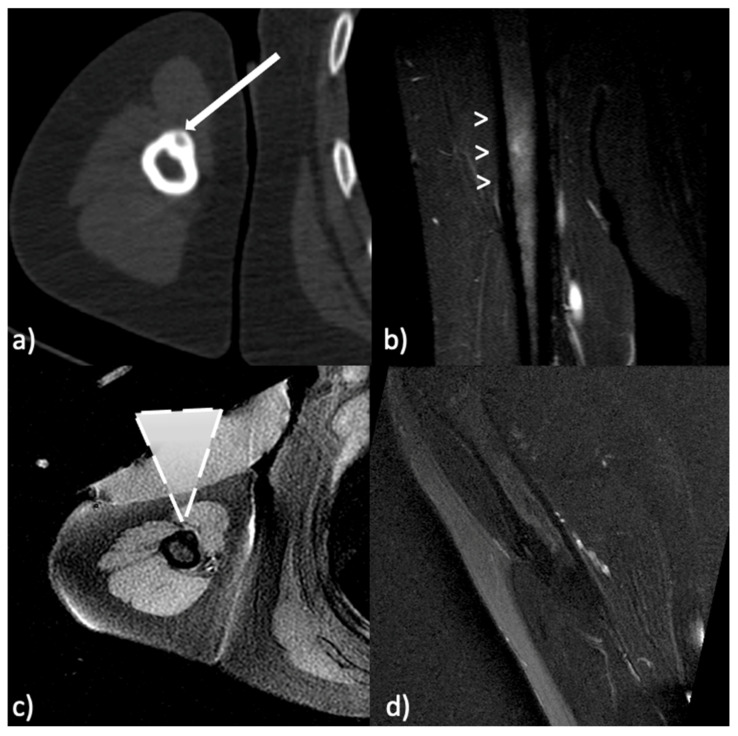
Osteoid osteoma of the omerus. (**a**) Pretreatment computed tomography (CT) shows the nidus (arrow) of the osteoid osteoma. (**b**) Pretreatment T2-weighted fast spin-echo magnetic resonance (MR) with fat-saturation shows bone marrow edema around the nidus (arrowheads). (**c**) MR image during treatment with the ultrasound (US) transducer in contact with the skin (dashed triangle represents the US beam focused on the target). (**d**) 1-year follow-up T2-weighted fast spin-echo MR with fat-saturation shows resolution of the bone edema.

**Figure 2:**
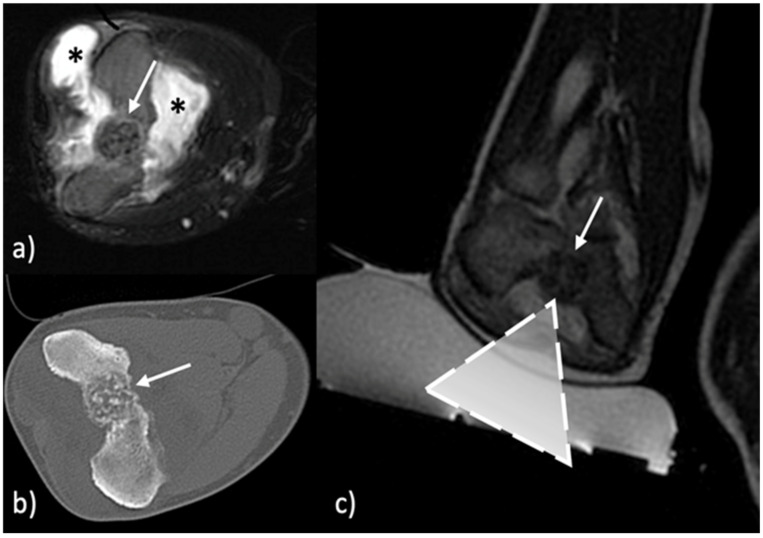
Intra-articular osteoblastoma of the elbow (**a**) Pretreatment T2-weighted fast spin-echo MR with fat-saturation shows the lesion (arrow), bone edema and the reactive intra-articular fluid (asterisk). (**b**) Pretreatment CT shows the nidus (arrow) of the osteoblastoma. (**c**) MR image during treatment with the US transducer in contact with the skin (dashed triangle represents the US beam focused on the target indicated by the arrow).

The presence of neurovascular structures along the pathway of the US beam is not a limitation when using MRgFUS, since the US beams pass through these structures without damaging them. Differing from the needle-based techniques (like RFA), when using MRgFUS it is not possible to move away critical structures using hydrodissection because there is the risk of injecting microbubbles of air in the area of treatment, and so the effectiveness of the procedure could be affected. In case critical structures are strictly closed to the target lesion (touching them or at the distance of a few millimeters), an accurate control of the temperature is needed to avoid thermal injuries. This may also be applied when tendons are close to the lesion. In Figure 1 and Figure 2, along the pathway of US beam and in the closeness proximity to the lesions there are no sensitive or critical structures that could be damaged by the heat.

The proper acoustic field is a key point for the clinical success of the treatment and is defined by these findings:

- Cortical or subperiosteal location of the lesion which must not be deeper than 2/3 mm in the cortex;
- Absence of interfaces between skin surface and lesion;
- Absence of any obstacles between skin and lesions

Another crucial aspect in planning MRgFUS is patient positioning, as it allows the US beam to optimally reach the target area. It is important that the skin above the lesion is in close contact with the transducer [26].

Different anesthesiologic approaches are used, depending on the location of the lesion, whose ablation is painful. Patients with lesions located in the upper arms are administered US-guided peripheral nerve block, whereas for lesions located in the hip joint, spinal anesthesia is used. After treatment, the patient recovery time is minimal (average time of 24 h) and an additional cortisone therapy is administered during the following week [31].

## 2. Literature and State of the Art

When applicable, MRgFUS is considered the first-choice treatment of benign bone tumors, due to absence of skin incision and ionizing radiation exposure [31].

Patients with non-spinal OOs are those who benefit more from this needleless approach.

Nevertheless, there is scarce literature describing features and treatment of benign bone lesions other than osteoid osteoma.

After an initial experience in 2013 based on six non-spinal OOs, all successfully treated in terms of alleviation of pain, and without complications [32], a larger and multicentric prospective study was carried out: 29 cases of non-spinal OOs [29] were treated and at 12 months of follow-up, complete clinical success (defined by absence of pain and interruption of analgesics) was obtained in 90%. Partial success was recorded in 10% of patients. In cases of complete clinical success, at 12-month imaging follow-up, a marked reduction in perilesional bone marrow edema and nidus vascularization was registered. No complications related to treatment were observed, demonstrating good accuracy and high safety of MRgFUS [29]. From these experiences it emerges how an accurate selection of patients is important as well as a comfortable patient position to achieve immobility.

Bazzocchi et al. [26] described 7 cases of superficial OOs of the lower limb treated with MRgFUS. During 12 months of follow up, no severe events or recurrences were registered. Primary outcome was pain relief: the Visual Analogue Scale (VAS) dropped to 0 after 1 month. In six cases, VAS remained at 0 during the study period, while in 1 patient VAS dropped from 9 to 0 after 1 month, rising to 2 after 3 months. When the clinical response in MRgFUS was compared to that of a cohort treated with CT-RFA, the clinical results were almost the same.

In line with literature, which describes good clinical response in terms of lack of recurrence in the long-term follow-up, our experience confirms a positive outcome of MRgFUS. We compared two groups of 15 patients, each affected by non-spinal OO. One group was submitted to MRgFUS and the other to CT-RFA. No significant differences were found in the primary and secondary outcome measures in terms of pain relief and recovery rate of compromised motor function. Complete response was obtained in 94% (MRgFUS) and 100% (RFA) of patients. No major complications were observed in both groups. The retrospective analysis on the 3 cases of 15 treated with MRgFUS resulted non-respondent. It was found that they presented a thick cortex and a thick layer of subcutaneous soft tissues. These results underline how the therapeutic success of MRgFUS is strictly dependent on the correct selection of patients [33].

More recently a bicentric retrospective study was conducted, analyzing 116 patients who underwent either CT-RFA or MRgFUS, over a mean of 2 years of follow-up [34]. This bicentric study confirmed that CT-RFA and MRgFUS were equally safe and effective in the treatment of OO. It was stressed that MRgFUS requires a strict anatomical selection to guarantee good results. In particular, a lesion is suitable for MRgFUS when it is well exposed to the US beam penetration with and adequate acoustic window. In this way, patients with scars or metallic devices which can interfere with the transmission of US beam are excluded. Similarly, OOs located deeply in the bone (intramedullary or with a thick layer of reactive bone) are not suitable for MRgFUS. Furthermore, this treatment has the benefit of treating larger areas with less invasiveness in presence of lesions on the bone surfaces.

Another important aspect is the treatment of bone lesions in pediatric population, where an ionizing radiation-free technique is particularly attractive. A recent multicentric experience in children (age <18 years) reported results on 33 OOs, performed in three centers, with 24 months of follow-up. The clinical outcome showed a primary success of 97%. One patient repeated the treatment obtaining success. No major complications were recorded. During the follow-up period no clinical relapses or further complications were observed. It was demonstrated that MRgFUS for OO has a clinical response comparable to CT-RFA, with the benefit of being a less invasive, ionizing radiation free technique [12].

To the best of our knowledge, when considering the application of MRgFUS for OBs, literature is very scarce. The larger of these published studies is a single-center retrospective analysis on 6 cases of intra-articular OBs, based on 2-year follow-up. The minimally invasive nature of MRgFUS was confirmed, and the absence of chondral or subchondral damage was reported. The authors also reported good results in terms of pain relief and imaging analysis, showing reduction of bone edema and synovitis with complete resolution. Conversely, they found a lack of significant correlation between VAS-determined pain change and VAS-determined functional impairment, perhaps because pain resolution does not mean complete recovery. This suggests the need of physical rehabilitation particularly in those cases where a long time elapsed between the onset of symptoms and the treatment [35].

In this scenario, approaching lesions located in the axial skeleton represents a crucial issue, as there is the risk to damage the bone marrow by heat conduction through the vertebral bone. OOs of the spine still represent a difficult treatment location through heat-based approach, mostly MRgFUS. The standard of care for these lesions is percutaneous RF or laser fiber ablation, which allow for a more accurate control of the volume of ablation and heat spread.

## Data Availability

The data presented in the study were obtained from the included trials and are openly available.
